# High-Accuracy Clock Synchronization in Low-Power Wireless sEMG Sensors

**DOI:** 10.3390/s25030756

**Published:** 2025-01-26

**Authors:** Giorgio Biagetti, Michele Sulis, Laura Falaschetti, Paolo Crippa

**Affiliations:** DII—Dipartimento di Ingegneria dell’Informazione, Università Politecnica delle Marche, Via Brecce Bianche 12, 60131 Ancona, Italy; s1125462@studenti.univpm.it (M.S.); l.falaschetti@univpm.it (L.F.); p.crippa@univpm.it (P.C.)

**Keywords:** biomedical sensors, Bluetooth Low Energy (BLE), clock synchronization, low power, real-time systems, surface electromyography (sEMG), synchronous data acquisition, wireless body sensor networks

## Abstract

Wireless surface electromyography (sEMG) sensors are very practical in that they can be worn freely, but the radio link between them and the receiver might cause unpredictable latencies that hinder the accurate synchronization of time between multiple sensors, which is an important aspect to study, e.g., the correlation between signals sampled at different sites. Moreover, to minimize power consumption, it can be useful to design a sensor with multiple clock domains so that each subsystem only runs at the minimum frequency for correct operation, thus saving energy. This paper presents the design, implementation, and test results of an sEMG sensor that uses Bluetooth Low Energy (BLE) communication and operates in three different clock domains to save power. In particular, this work focuses on the synchronization problem that arises from these design choices. It was solved through a detailed study of the timings experimentally observed over the BLE connection, and through the use of a dual-stage filtering mechanism to remove timestamp measurement noise. Time synchronization through three different clock domains (receiver, microcontroller, and ADC) was thus achieved, with a resulting total jitter of just 47 µs RMS for a 1.25 ms sampling period, while the dedicated ADC clock domain saved between 10% to 50% of power, depending on the selected data rate.

## 1. Introduction

The aging of the world population highlighted in recent years by the “World Population Prospects” published periodically by the United Nations [1], along with advances in cutting-edge electronics, signal processing, and wireless networks for 5G and the upcoming 6G communication technologies, have pushed research to revolutionize traditional healthcare methodologies and assistive technologies [2,3]. By implementing these technologies, patient monitoring procedures can be performed remotely, from home or during normal daily activities, with the virtual supervision of doctors, therapists, healthcare professionals, or trainers. Accordingly, wearable medical devices should be comfortably worn by patients to monitor their daily living activities without interfering or limiting the patient’s normal range of action/motion or their daily routine. Biomedical sensors are key components of these wearable medical devices and are basically physical enablers, which play a vital role in any eHealth architecture. Physiological signals are acquired from various sensors such as electrocardiography (ECG), electromyography (EMG), photoplethysmography (PPG), electroencephalography (EEG), phonocardiography (PCG), and inertial measurement units (IMUs). Surface electromyography (sEMG) measures and records the electrical activity of muscles near the surface of the skin by acquiring the electrical voltage across a pair of electrodes placed on the skin over the muscle of interest. By analyzing sEMG signals, it is possible to evaluate the contraction and relaxation of different muscles and thus discriminate the gestures performed by the patient [4]. Since sEMG signals are weak, with a low-level amplitude (fractions of millivolts) and a high-impedance source (up to tens of kiloohm), they are not so easy to acquire and need sophisticated and ad hoc circuitry [5,6].

In the last five years, the use of signals acquired from muscles has become of critical importance also thanks to the progress of embedded systems and machine learning techniques that exponentially increased the amount of useful information obtainable from them. Often, this information is obtained by deploying several wireless tiny sensors across the body, creating so-called Wireless Body Area Networks (WBANs), or in the case of one or more base stations for collecting data, a more general Wireless Sensor Network (WSN). In this scenario, ensuring synchronization between devices is a significant issue due the existence of the multiple clock sources in the sensor nodes [7,8]. Indeed, if each sensor uses its own oscillator as the time base, these time scales will inevitably drift apart over time due to unavoidable frequency differences in the local oscillators. Standard crystals used in these oscillators can have baseline frequency tolerances of the order of 50 ppm (tighter tolerances of around 10 ppm exist, but are more expensive), to which aging drift and temperature dependence should be added. Such a tolerance might seem small but will add up to a drift of 1/10th of a second in just half an hour, making long-term measures incoherent. Because of this, time scale alignment between sensors is a non-negotiable requirement, and it can be achieved by either synchronizing the frequencies of the oscillators or by re-scaling the time base of the acquired signals.

Several techniques for clock synchronization in WSNs and WBANs were proposed to ensure the synchronization of sensor data obtained from different nodes [9]. However, this has become a critical task in specific applications of WBANs, e.g., in human activity recognition or in rehabilitation biomedical systems, which require high data acquisition sampling rate, long-duration continuous sampling, multi-device synchronous measurements, and data fusion, as well as low power consumption.

A large variety of possible solutions to ease the adoption of these sensors have been proposed in the literature. In particular, transforming existing sEMG measurement systems into portable wireless and wearable devices to assess muscle strength is key for digital healthcare and rehabilitation applications such as robotic limb or exoskeleton control as in [10], where a two-channel EMG signal wireless acquisition system was proposed for human lower limb activity recognition. In [11], an sEMG acquisition system that combines a graphene-based flexible electrode with a signal acquisition flexible printed circuit board was proposed for assessing muscle strength and hand rehabilitation training using a long short-term memory network training model. Existing prototypes of capacitive EMG (cEMG) sensors are typically large and designed with a hybrid printed circuit board (PCB). In [12], a fully flexible cEMG biomedical sensor with integrated front-end analog circuitry in a single medical plaster-sized device was proposed. Similarly, in [13], a flexible capacitive multi-channel biomedical wireless acquisition system was proposed. The system consists of flexible cEMG sensors, a low-noise wireless data acquisition module, and post-signal processing algorithms.

Furthermore, wearable devices that integrate multiple sensors capable of simultaneously acquiring inertial and different physiological data have emerged. In [14], a wearable device that combines the acquisition of EMG signals from the cervical region with inertial data from IMUs to assess the occurrence of a forward head posture condition was presented. As a further example, in [15], a wireless multilayered sensor that can simultaneously acquire sEMG, mechanomyography (MMG), and near-infrared spectroscopy (NIRS) signals was proposed to evaluate muscular activity and fatigue during exercises. In [16], the design, implementation, and evaluation of a device that performs EMG and electrical impedance myography (EIM) simultaneously allowed a more robust measurement of muscle conditions subject to artifacts. Alternatively, acquiring both ECG and sEMG signals along with inertial data is essential to efficiently treat and effectively expedite motor recovery in cardiac patients after a stroke. The authors in [17] presented a wireless ECG/EMG signal acquisition device and a smartphone-based software platform for real-time data processing and monitoring, and cloud server access for 24/7 patient monitoring. Interference noise that can occur during a regular at-home environment was eliminated through adaptive digital filtering while minimizing the data processing time.

In order to deploy multiple sensors at a low cost with low latency of the acquired data, Bluetooth Low Energy (BLE) was often chosen as the wireless communication protocol, as it was deemed more suitable than ZigBee or low-power Wi-Fi for internal communication in WBAN star or mesh topologies, such as those used for multi-channel wireless sEMG-based biomedical systems [18,19]. However, current time synchronization methods for BLE multi-channel systems, via either BLE beacon transmissions or additional hardware, can hardly satisfy the requirements of high throughput with low latency, transferability between commercial devices, and low energy consumption. In [20], time synchronization and data alignment algorithms were implemented and tested in the BLE application layer without the need for additional hardware. Similarly, in [21], a low-latency, BLE-based data alignment method was implemented in the BLE application layer.

Of course, other protocols can be used besides BLE to also achieve synchronization. Examples include [22], where a fully digital circuit for one-way master-to-slave, highly precise time synchronization in a low-power wearable system equipped with a set of sensor nodes is described. These sensors are wired together to form a mesh, using conductive yarns to communicate. The circuit performs synchronization at the MAC layer. In each sensor node, the synchronization circuit provides a programmable clock signal and a real-time counter for timestamping. In [23], a platform composed of an IMU and an sEMG unit, which is able to store raw data or on/off muscle activation information, was proposed. The system uses an ad hoc low-complexity synchronization protocol based on a master–slave architecture that guarantees synchronization of measurement, even during long-lasting acquisitions. In [24], an original wireless joint communication and localization EMG-sensing concept was proposed. An on-body sensor beacon measures EMG signals and wirelessly transmits them. At the same time, the spatial position and movement of the beacon are determined with high precision in real time using these transmitted radio signals. This avoids the need to explicitly synchronize multiple independently operating sensors, without using complex time synchronization protocols. In contrast, in [25], data synchronization accuracy and low power consumption were ensured for real-time synchronous data acquisition using a proprietary protocol based on time-division multiple access (TDMA), which enables very precise timing to be achieved and incorporates deep energy-efficient coding in the sensor firmware.

This work presents the design of an sEMG sensor based on [26]. To further optimize power consumption, an additional clock domain dedicated to the analog-to-digital converter (ADC) was added. This improves the circuit performance in two aspects: (i) reduces the period jitter of the clock used by the sigma–delta modulator in the ADC, thus potentially lowering the noise floor of the converter, and (ii) decreases the power consumption by allowing the usage of a lower-frequency oscillator for the ADC, instead of deriving its clock from the high-frequency oscillator used by the BLE radio. This led to the necessity of upgrading the synchronization scheme to take into consideration frequency differences between these internal clocks in addition to the differences between devices. To solve the problem, a technique to synchronize, with an absolute time scale (e.g., UTC, or what is commonly called “wall clock”), the data sampled by wireless sEMG sensors and transmitted through a variable-latency radio link such as BLE, is described. This technique falls within the post-acquisition time scale adjustment category, and was preferred over real-time oscillator frequency adjustment because it does not require extra hardware on the sensor nodes, thus keeping their cost and energy consumption low, while still being able to operate with very low latencies. It provides very high timing accuracy and is easy to implement, while minimizing the power consumption on the wearable sensor node, by performing all the required computation on the receiver.

This paper is structured as follows: Section 2 describes the system hardware and presents some data to highlight details of the synchronization problem, together with the filters and algorithms used to solve it. Section 3 presents the experiments performed to validate both the power consumption and the timing accuracy achieved by the presented system. Section 4 discusses the obtained results in the light of the state of the art, while Section 5 draws some conclusions and outlines possible future improvements.

## 2. Materials and Methods

**Figure 1:**
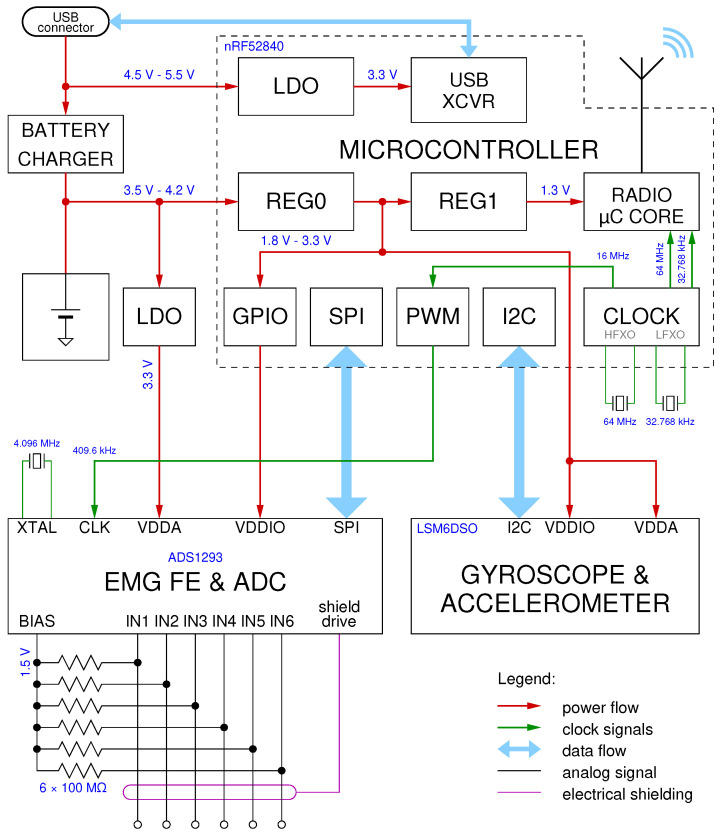
Block diagram of the sEMG sensor. It is based on a Nordic Semiconductor nRF52840 SoC that integrates an ARM Cortex-M4 CPU running at 64 MHz and a multi-protocol radio compatible with Bluetooth 5 Low-Energy mode. A Texas Instruments ADS1293 integrated analog front-end (AFE) and ADC are at the core of the sEMG signal acquisition chain, while an STMicroelectronics LSM6DSO inertial measurement unit complements the system.

The sEMG sensor considered in this work is an evolution of the one presented in [26]. There, a new combined sEMG and inertial sensor was described, specifically designed for long-term use, with a particular emphasis on low power consumption. The block diagram of the updated version is shown in Figure 1.

In the original design, some compromises were made to reduce production costs, and to simplify data acquisition. For instance, the sEMG acquisition chain employs a Texas Instruments ADS1293 analog front-end with an integrated ADC converter. The sigma–delta modulator within its ADC stage requires a 409.6 kHz clock signal for optimal performance, and in the original design, this was derived from the main microcontroller crystal oscillator (64 MHz) through a fractional divider, using a suitable waveform-driven PWM modulator in the microcontroller.

This design decision allowed a very strict relationship between microcontroller time and ADC sampling, thus allowing relatively easy timestamp reconstruction at the receiver side, as demonstrated in the aforementioned paper. But it also had some drawbacks, the most obvious of which is increased power consumption due to the always-on operation of the high-frequency oscillator (HFXO) and PWM in the microcontroller, together with the DMA engine to fetch the PWM waveforms from RAM for the fractional division.

Moreover, usage of the fractional divider to obtain 409.6 kHz from a 16 MHz signal (the PWM modulator works from a divided-by-4 peripheral clock derived from the CPU clock, so we need to divide by 39.0625=39+1/16) leads to additional period jitter in the ADC clock (which is a well-known cause of increased noise in delta–sigma modulators [27]).

To try and alleviate these two drawbacks, the updated design added a dedicated 4.096 MHz crystal to the AFE. This additional clock source inevitably leads to a loss of the perfect synchronization between the AFE and microcontroller, so the timestamp reconstruction algorithm had to be rewritten to take into account yet another time scale, and the new algorithm and its performance is the focus of this paper.

### 2.1. Jitter Reduction

The period jitter is defined as the root mean square (RMS) of the difference between the actual clock period and the ideal clock period. Due to unavoidable inaccuracies in the oscillator frequency of both the device under test and the measurement instrument, the ideal clock period is commonly assumed to be equal to the average period over a large number of measurements, so that the jitter can be estimated as the standard deviation of the measured clock periods. JEDEC standard JESD65B recommends to average over 10,000 randomly selected clock periods. Letting $T_{j}$ be the *j*-th measured clock period, and $T_{0}$ its average or nominal value, the period jitter is then defined as

(1) $$t_{\mathrm{jit}(\mathrm{per})}=\sqrt{\frac{1}{N}\sum_{j=1}^{N}{\left(T_{j}-T_{0}\right)}^{2}}$$

where *N* is the number of periods considered. For our fractional divider, since it ideally produces a perfectly periodic sequence that repeats every 16 clock cycles, we can assume N=16, $T_{j}=39\;T_{\mathrm{PWM}}$ for j∈{1,⋯,15}, $T_{16}=40\;T_{\mathrm{PWM}}$, with $1/T_{\mathrm{PWM}}=16\;\mathrm{MHz}$, which yields $T_{0}=\left(15\times 39+40\right)/16\;T_{\mathrm{PWM}}$ as expected, and an ideal $t_{\mathrm{jit}(\mathrm{per})}$ of about 15.129 ns RMS. Since the actual measured total period jitter on a sample prototype was 15.135 ns (see Section 3), this fractional divider is clearly the major cause of clock uncertainty in the sigma–delta modulator.

The re-designed sensor node uses a crystal oscillator directly driven by the ADC chip. It required the addition of a relatively large 4.096 MHz crystal to the sensing node (unlike their higher-frequency and lower-frequency counterparts, crystals at this particular frequency range are generally not available in small form factors), as the ADC chip has an integrated divide-by-10 pre-scaler to derive its modulator clock, but in the end, we managed to squeeze it in while maintaining the same PCB sizes, so that the nodes would still fit within the same enclosures.

**Figure 2:**
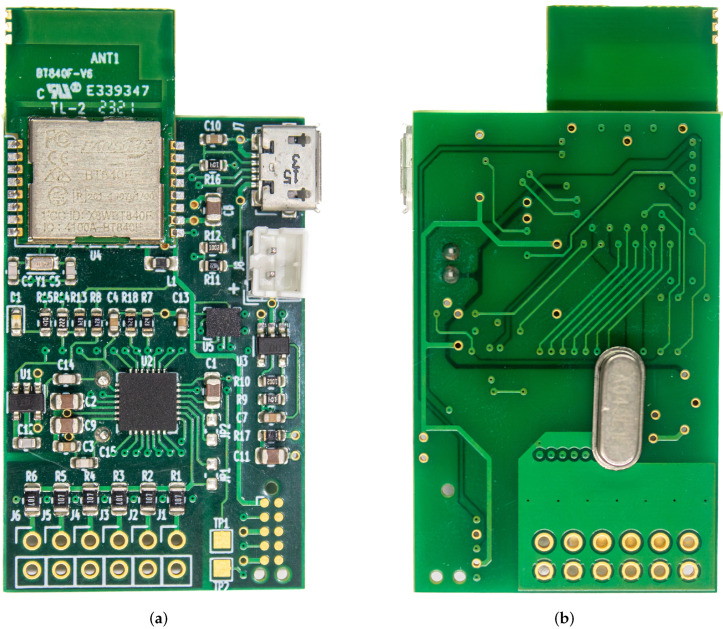
Pictures of the assembled prototype (scale 2:1). (**a**) Front side: The ADS1293 AFE (U2) is clearly visible in the center, the BLE radio and microcontroller at the top (beneath the RF shield), and pads for the electrodes at the bottom. (**b**) Back side: The 4.096 MHz crystal was added here as there was no room on the front side. Being a THT component, it was bound to be soldered manually anyway, so this type of double-sided assembly did not significantly increase production costs.

A picture of the re-designed prototype sensor node can be seen in Figure 2; the PCB still measures 51mm×27mm. Three pairs of (preferably shielded) electrode cables can be connected to pads J1–J6.

As could be expected, the jitter was dramatically reduced by avoiding the use of a fractional divider, and the measured clock jitter on the 409.6 kHz signal derived from the crystal oscillator was just 0.139 ns RMS, which is comparable to the measurement uncertainty of our jitter estimation setup, as will be discussed later.

### 2.2. Measuring Time on Multiple Clocks

Having different clock sources for the ADC and microcontroller, on the other hand, meant that the timestamp reconstruction and synchronization method described in [26] could no longer be applied as-is to the new sensor node, as the packet transmission timestamps (sampled from a microcontroller timer) are no longer synchronous to ADC sampling.

The synchronization problem can be stated as follows: We assume that the BLE receiver (which can be a PC or a smartphone) has a clock source already synchronized to some absolute time scale via external means, e.g., by the Network Time Protocol (NTP), or even by the Precision Time Protocol (PTP) if better accuracy is deemed necessary. We will call this absolute time scale the receiver time scale, and denote as $t_{\mathrm{RX}}$ time samples measured along said time scale.

The sensor node has two other different time scales: the microcontroller time scale, and the ADC time scale. The analog signal is “sampled” at integer multiples of the selected sampling period according to the ADC time scale. The acquired data are then buffered multiple times: at the firmware application level, to fill packets according to the format requested, and then at the Bluetooth stack level, where packets are queued awaiting connection events from the central (receiver) before being transmitted. The timing of the transmission over BLE is, indeed, entirely governed by the central, which periodically transmits beacons to the connected peripherals to query whether they have data to transmit (or to send data or commands to them if need be). So, even if the protocol we designed uses notifications to stream data, as they are the lowest-latency mechanism in BLE, they are still queued and delayed till the next connection event, when a short burst of notifications is eventually transmitted. Also, RF interferences or channel congestion can cause a connection event to be cut short or even skipped altogether, so the actual latency is not predictable.

To try and minimize the impact of this Bluetooth queuing mechanism, the node firmware was designed to configure the radio to timestamp actual packet transmission time (after queuing) of the packets sent. The Nordic Semiconductor nRF52840 can achieve this in hardware, so that there are no latencies due to interrupts and software execution speed, but of course, these timestamps follow the microcontroller time scale. We will denote them as $t_{\mathrm{TX}}$.

The buffering performed by the firmware must also be taken into account. To this end, the ADC sampling time is also timestamped in hardware by the microcontroller before SPI transmission from the ADC even takes place. We will denote these timestamps as $t_{\mathrm{AD}}$, always referred to the microcontroller time scale. Please note that in this time scale, $t_{\mathrm{AD}}$ will not be evenly spaced, as they would have been if the ADC time scale had been used, or if the ADC time base had been derived from the microcontroller time base as we did in [26].

**Figure 3:**
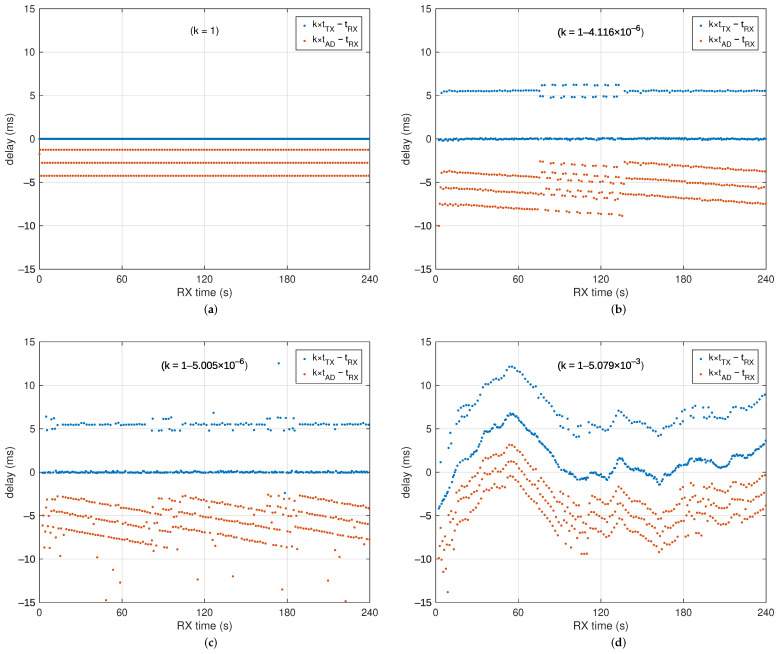
Examples of measured timestamps. The microcontroller time scale is corrected (to take into account oscillator frequency error) by multiplying it by a constant *k* obtained by linear regression with the RX time scale, so that the curves fit within the graph even for large deviations of *k* from 1. As a consequence, the blue dot “lines” appear almost horizontal, even though their actual slope (frequency error) should be 1/k. (**a**) Ideal results as obtained from high-level VHDL simulation of the system, (**b**) measured differences using the 64 MHz HFXO to clock the microcontroller and the PWM fractional divider to clock the ADC, (**c**) measured differences using the 64 MHz HFXO to clock the microcontroller and the independent 4.096 MHz crystal to clock the ADC, (**d**) measured differences using the internal RC 64 MHz low-power oscillator to clock the microcontroller and the independent 4.096 MHz crystal to clock the ADC.

To better highlight how these time scales relate to each other and the problems that need to be solved to align them, some examples are reported in Figure 3, which reports differences between the transmitted timestamps $t_{\mathrm{TX}}$ (in blue) and $t_{\mathrm{AD}}$ (in red) with respect to the reception time of the same packet $t_{\mathrm{RX}}$. Experiments were performed using (**a**) a high-level VHDL model of the buffering mechanism of BLE, to see what could be expected in an ideal situation, (**b**) with the clocking scheme as in [26], which uses the same time scale for both $t_{\mathrm{TX}}$ and $t_{\mathrm{AD}}$, (**c**) activating the 4.096 MHz crystal for the ADC (while keeping the 64 MHz crystal on), (**d**) and finally de-activating the 64 MHz crystal when not needed as the ADC has its own clock source (the microcontroller continues to run with its internal low-power RC oscillator, which, of course, has poor accuracy and stability).

To save bandwidth, the timestamps are only transmitted at a low bit-rate in so-called subchannels, once every 256 packets. The examples in the aforementioned figure are obtained with the default 3-channel, $F_{S}=800\;\mathrm{Hz}$, 24-bit ADC setup, which means every packet contains $N_{S}=2$ time samples for each channel, for a total of 18 bytes of payload, and that it takes a time $T_{\mathrm{TS}}=256\;N_{S}/F_{S}=640\;\mathrm{ms}$ to fill up 256 packets. Using the minimum BLE connection interval (CI) $T_{\mathrm{CI}}$ of 7.5 ms, nominally, a burst of $N_{P}=F_{S}\;T_{\mathrm{CI}}/N_{S}=3$ packets per CI is sent. So, since TTS/TCI=85+13, the dots representing the $t_{\mathrm{AD}}-t_{\mathrm{RX}}$ difference align over three different bands (lines, ideally) according to the position within the burst, as the buffering delay from sampling till the next CI varies accordingly.

The dots representing the $t_{\mathrm{TX}}-t_{\mathrm{RX}}$ difference should in theory all align over a single line, but it has been observed that they distribute over (at least) two bands, in what looks to be an approximate 1:2 ratio when no other causes of error (like packet retransmissions) are present. The exact reasons under this distribution is not yet fully understood. TX timestamping is performed in hardware, so we have not much control over its actual behavior, but the distribution looks like an off-by-one error in the association of the recorded timestamp to the packet index. But it can also be caused by receiver-side buffering or other causes. We believe this is quite a seldomly used feature of this microcontroller that has not yet received much investigation. Anyway, since packets will be inevitably delayed when RF interferences are present and need to be retransmitted, we will treat this “band splitting” just like any other source of noise the synchronizer filter needs to remove. As for the slope of the “lines” formed by the $t_{\mathrm{AD}}-t_{\mathrm{RX}}$ bands, in (**b**), they are due to the frequency error of the receiving BLE radio that uses a $T_{\mathrm{CI}}$ not exactly equal to 7.5 ms, while in (**c**), they are also affected by the frequency difference between the microcontroller HFXO and the ADC crystal.

### 2.3. Synchronization

The main objective to achieve is to avoid the long-term drift of time scales, so that measures made from multiple sensors can be properly aligned. Referencing captured data to an absolute time scale is probably the most practical way to achieve this, as it is already an agreed-upon time scale. But as shown in the previous subsection, the delay between the sampling of data and reception, while following a somewhat logical scheme, is hardly predictable with any precision because of buffering delays.

So, we envisioned a two-step scheme: first, the microcontroller time scale is aligned with real time. This, at least when the HFXO is used, is almost a straight line, provided artifacts are excluded. Then, the sample index is related to the microcontroller time. Overall, the process can be split by estimating two functions:

(2) $$f_{\mathrm{TX}}\to \mathrm{RX}:t_{\mathrm{TX}}\mapsto t_{\mathrm{RX}}$$

(3) $$f_{n}\to \mathrm{AD}:n\mapsto t_{\mathrm{AD}}$$

where *n* is the sample number for which we wish to estimate the sampling time. In general, these functions will need to be time-varying to follow crystal drift due to aging and temperature variations, but it is reasonable to assume these variations to be slow compared to the sampling frequency, and so a first-order spline model for them can be used. It is equivalent to performing a local linear regression.

Estimating (2) is not as easy as it can appear, because the occasional retransmissions pose quite a significant challenge. To avoid these, we adopted a statistical filter that estimates the histogram of the distribution of the delays $d=t_{\mathrm{TX}}-t_{\mathrm{RX}}$ and discards the points that are too far away from the mode. This is achieved by quantizing *d* to millisecond resolution, and then counting the number of occurrences of each difference so that the most frequent value (mode) can be estimated. Data points deviating by more than 2.5 ms from the estimated mode are then excluded from regression fitting.

The remaining points can be used to fit a linear model to (locally) express $t_{\mathrm{RX}}$ as

(4) $$t_{\mathrm{RX}}=b_{1}\;t_{\mathrm{TX}}+b_{0}$$

with $b_{1}$ and $b_{0}$ being the regression coefficients.

Estimating (3) should be easier because it is not affected by buffering delays or interferences. Again, a locally linear model such as

(5) $$t_{\mathrm{AD}}=c_{1}\;n+c_{0}$$

is fitted to the data, with $c_{1}$ and $c_{0}$ being the regression coefficients. Please remember that $t_{\mathrm{AD}}$ is known only for values of *n* that are integer multiples of 256 (and that there can be missing samples), so that the regression is also used as an interpolator.

Since $t_{\mathrm{AD}}$ and $t_{\mathrm{TX}}$ are measured from the same time base (microcontroller clock), it is possible to combine functions (3) and (2) to map the index of any received sample *n* to a time *t* in the receiver time scale, $t=b_{1}\;\left(c_{1}\;n+c_{0}\right)+b_{0}$, which accomplishes the desired goal of associating an absolute timestamp to each received signal sample. This allows accurate compensation of oscillator drift, as will be shown in the next section.

## 3. Results

**Figure 4:**
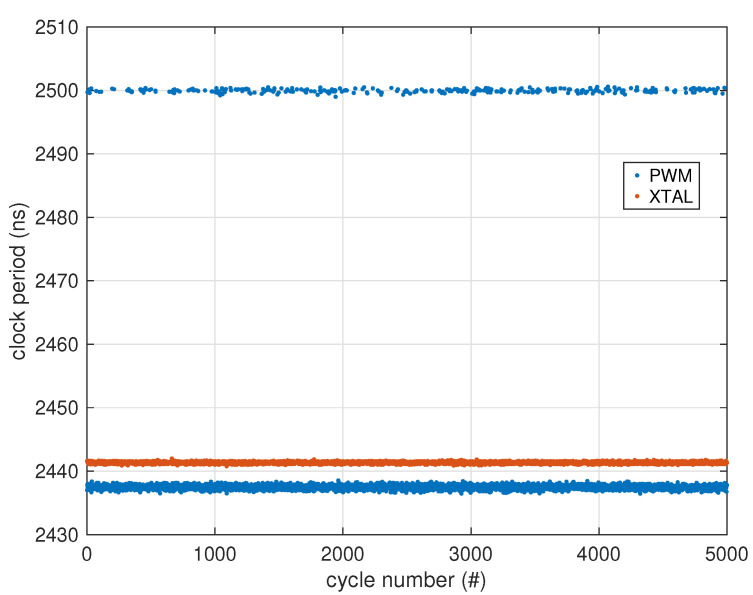
Jitter measurement. The blue dots represent clock periods measured with the PWM output (fractional divider); 1/16th of them are around 2500 ns ($40\;T_{\mathrm{PWM}}$), and 15/16th of them around 2437.5 ns ($39\;T_{\mathrm{PWM}}$). The red dots are measured with the 4.096 MHz crystal oscillator output internally divided by 10 to produce the required 409.6 kHz (2441.4 ns) modulator clock.

The first test performed to assess the effectiveness of the additional crystal added to the circuit is an estimate of the delta–sigma modulator clock jitter. Figure 4 shows a portion of the measured clock periods with the fractional divider active (marked as “PWM”), and with the crystal oscillator active (marked as “XTAL”).

The clock periods were measured using a PicoScope 3203D MSO (Pico Technology, St Neots, Cambridgeshire, UK) mixed-signal oscilloscope, averaging 1000 waveforms sampled at 1 GS/s for a duration of 50 µs each, for a total of 20,000 clock cycles analyzed. Each clock period was detected using a 1.5 V threshold with linear interpolation of the two closest samples, since the digital supply voltage was set to 3.0 V for this experiment. Results are reported in Table 1. The instrument has a ±50 ppm time base accuracy with a typical sample jitter of ±3 ps RMS, with a vertical axis noise of about ±1 LSB and an LSB of 47.213 mV at the selected range. Given that the clock edges from the PWM output had a measured slew rate of 258 mV/ns and those from the crystal oscillator had a slew rate of 425 mV/ns, which leads to a measurement uncertainty of the threshold crossing point of about 0.111 ns, the jitter figure obtained for the crystal oscillator is clearly dominated by measurement noise and can be expected to be much lower than that.

The different clock sources can also be confirmed by analyzing the statistics of the difference between consecutive sampling timestamps $t_{\mathrm{AD}}$, denoted as $\Delta t_{\mathrm{AD}}$. Since they are transmitted every 256 packets, i.e., every 512 samples at an $F_{S}=800\;\mathrm{Hz}$, if the ADC is synchronous to the microcontroller, it must be exactly $\Delta t_{\mathrm{AD}}=640,000\;\mu s$, given that the resolution of the timestamps is 1 µs. This was confirmed experimentally on all the prototypes tested. On the other hand, enabling both crystals, $\Delta t_{\mathrm{AD}}$ varied (for instance, on a sample prototype) between 639,982 µs and 639,983 µs, with a long-term average of 639,982.9 µs, denoting a 17 ppm frequency deviation from the nominal value of the two oscillators, perfectly within the stated tolerances.

To test the effectiveness of the synchronization strategy, a pulse train was fed to the EMG sensor. The pulse train was generated by a Raspberry Pi 4 toggling a GPIO high for 10 ms every 29.9 ms. The software control continuously polled the real-time clock, which is synchronized to UTC via NTP, to set the state of the GPIO pin. This avoids the drift and frequency errors of the internal oscillators, at the cost of a little jitter due to software delays. The jitter in the generated pulse train was estimated to be 22 ns RMS, with a maximum deviation in the pulse edge from its ideal position of 94 ns, basically negligible since the EMG samples data at 800 Hz, i.e., with a 1.25 ms period.

**Figure 5:**
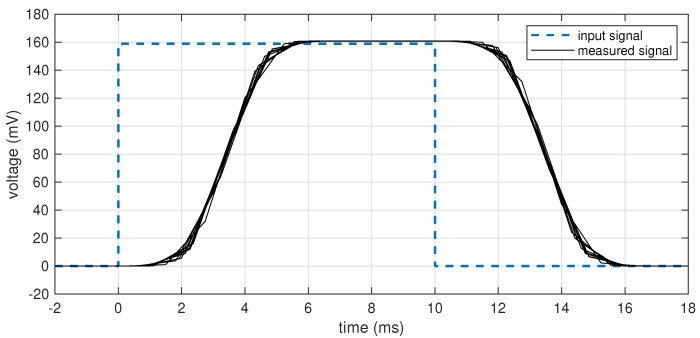
Synchronization of the received data to an absolute time scale. The time axis represents the absolute time (obtained through NTP) from the rising edge of the applied pulses (dashed blue line). The black lines are 10 randomly selected measures over a span of 2 min.

The data were transmitted over BLE and acquired by the same Raspberry Pi, so that the receiver time scale was the same as the time base for the pulse train generation. The acquired data were then processed according to the proposed dual-stage compensation, for a total of 10,000 pulses analyzed. Results for 10 randomly selected pulses are shown in Figure 5, whose time axis is set to 0 in correspondence to the ideal position of the rising edge of the pulse. The low-pass response is typical of the three-stage sinc^5^ filter included in the ADS1293 to decimate the sigma–delta modulator output. As can be seen, there is no noticeable time displacement of the measured pulse over its ideal position. The measured average period of the impulses was 29.900004350 ms, with an error of only 0.14 ppm, much lower than the crystal tolerances, and the total jitter (measured edge position vs. ideal edge position) was 47 µs RMS.

**Figure 6:**
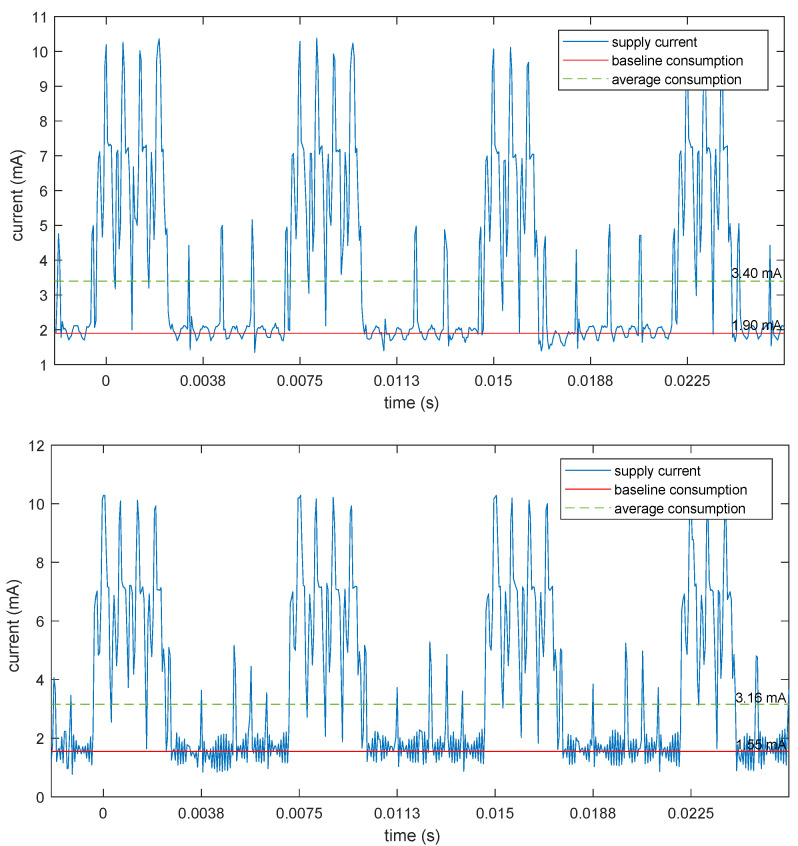
Measurement of the current drawn by the device while streaming data at 800 Hz, 3 channels, 24 bit per channel. Top: using the PWM fractional divider as the clock source for the ADC. Bottom: using the dedicated crystal for the ADC clock. Peaks correspond to packet transmission, while the clock source mainly affect the baseline draw and will thus be much more evident with lower rate configurations.

Finally, to confirm the power savings obtained by the usage of the additional crystal, a comparison of the current consumption profile between the two clocking methods (PWM and dedicated crystal) is shown in Figure 6. It reports the total device current drawn from a 3.7 V source (battery) while streaming data. Four connection intervals are shown, while the averages are computed over a 5 s time span.

**Figure 7:**
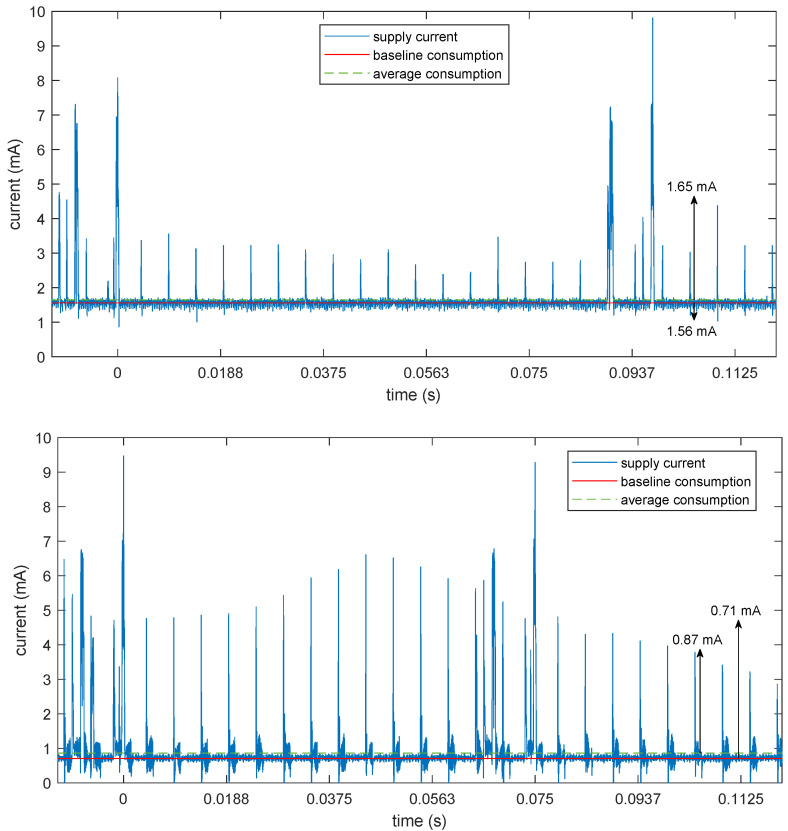
Measurement of the current drawn by the device while streaming data at 200 Hz, 1 channel, 20 bit per channel, VLDE encoding. Top: using the PWM fractional divider as the clock source for the ADC. Bottom: using the dedicated crystal for the ADC clock.

The energy saving is apparent in the baseline consumption, which was reduced from 1.90 mA to 1.55 mA, corresponding to a saving of about 18%. Of course, with such a high data rate, most of the power consumption is due to microcontroller activity and radio transmissions, subsystems that are unaffected by the additional clock domain. The energy saving will be much more apparent in a lower rate configuration. To show this, Figure 7 shows the same current traces for a configuration of the device with only one active channel, 200 Hz sampling (suitable, e.g., for a long-term ECG monitoring), 20 bits per sample, further losslessly compressed to 8 bits per samples in the transmitted packet through the use of a variable-length differential encoding (VLDE) scheme as described in [28]. This huge reduction in the number of bits can be achieved losslessly because most of the 24 bits (or 20 bits in this case) of the ADC are actually used to track the baseline wander of the signal due to the direct (dc) coupling of the electrodes. This can be a huge but slowly time-varying signal, which can be removed by differentiating the data stream before encoding. The differentiation process is perfectly reversible at the receiver side, so no information is lost here. Then, the differences can be encoded using fewer bits, just 8 in most cases, as demonstrated in the aforementioned paper.

With this lower data rate, transmission bursts only happen on average every 90 ms instead of every connection interval, and the microcontroller can spend much more time idling with the HFXO off while the ADC is being clocked by its dedicated oscillator. The average and baseline power consumptions are thus very similar, with the average current being reduced from 1.65 mA in the PWM case to 0.87 mA in the dedicated crystal case, corresponding to a reduction of 47%, almost one half of the total power.

## 4. Discussion

Adding a separate clock domain for the ADC converter can result in significant power savings when the microcontroller can be put to sleep for a significant fraction of the time, i.e., when it is not working at the maximum specified data rate. With a typical single-lead ECG configuration, this allows us to save almost 50% of the power, thus doubling the battery life and allowing long-term evaluations (over 10 days of continuous streaming with a small 240 mAh lithium-ion rechargeable battery). At the maximum rate, there is still a 10% reduction in power.

To better appreciate the possible savings, Table 2 reports a breakdown of the measured power consumption for the two considered streaming configurations and for the three different possible clocking schemes: using either the 64 MHz HFXO for the microcontroller or its internal RC oscillator, and driving the ADC with the PWM output from the microcontroller (which implicitly requires usage of the HFXO) or with its dedicated 4.096 MHz crystal.

As can be seen, switching the microcontroller clock source to the RC oscillator (when it is not busy transmitting data over the radio) provided additional energy savings compared to always keeping the HFXO on. This, on the other hand, results in an increased error in the absolute time synchronization as it is necessary to refer to its clock domain to convert ADC timestamps to receiver absolute time, from 6.6 µs achieved by us in [26] (mode XTAL + PWM) to 47 µs of the present work. Nevertheless, given the nature of the EMG signals, which are sampled at millisecond intervals, microsecond timing accuracy is still very tight and unlikely to be noticeable in any signal analysis.

To position the present result with respect to the state of the art, Table 3 reports a comparison of the achieved accuracy by other works in the literature, with a brief summary of the system configuration and protocol used.

As can be seen, most works used the Nordic Semiconductor family of microcontrollers, either the same SoC as us (nRF52840) or its previous generation (nRF52832). One work developed a custom radio protocol on top of it to achieve tighter synchronization, but other works using this SoC family over BLE obtained either much worse [21] or slightly better [23] performance. As a reference, a wired synchronization system was also included. Of course, without the delays of the radio modem, much tighter synchronization can be achieved at the nanosecond level, but that is also completely useless for EMG signals, and required the development of a custom ASIC.

## 5. Conclusions

In this paper, we presented a detailed study of the challenges regarding accurate clock synchronization in a wireless sEMG sensor that sends data over a BLE connection. Issues regarding the presence of multiple time scales due to usage of different crystals to optimally clock circuit subsystems have been presented, showing how they interact to the buffering inherent to any BLE-based transmission. Proper and accurate alignment of these time scales require adequate insertion and filtering of timestamps in packets, as the transmission delays cannot be totally deterministic due to unavoidable RF interferences, receiver-side packet processing and queueing between the OS and application layer, and possibly transmitter-side hardware timestamping errors. The major contributions of this work consist in the analysis of the distribution of actual timestamp data, which led to the development of a simple and yet effective statistical filtering technique, coupled with piecewise linear interpolation, to extract smooth and reliable estimates of wallclock-related ADC sampling timestamps, and in the analysis of the power savings that each clocking strategy can achieve under different usage scenarios. By using a dedicated crystal to clock the ADC and reverting to a less accurate but more energy-efficient RC oscillator to clock non-time-critical operations of the MCU, a nearly 50% reduction in power was achieved while streaming single-lead ECG data, demonstrating the possibility of building low-power, near-real-time, and high-accuracy BLE signal streaming systems.

Future research directions could include further investigation on the root causes of TX timestamping inaccuracies and/or errors, together with the analysis of the performance on an actual multi-channel setup.

## Figures and Tables

**Table 1:** Measured performance of the two studied timing sources for the sigma–delta modulator.

Timing Source	Measured Frequency (kHz)	RMS Period Jitter (ns)
PWM	409.604	15.135
XTAL	409.612	0.139 ^1^

^1^ The threshold crossing point uncertainty of this measurement setup is ±0.111 ns, so this figure is likely biased.

**Table 2:** Power consumption comparison of the different clocking schemes.

Clocking Scheme (MCU + ADC)	Power Consumption (High-Data-Rate Mode) ^1^	Power Consumption (Low-Data-Rate Mode) ^2^
XTAL + PWM	12.55 mW	6.07 mW
XTAL + XTAL	11.72 mW	3.70 mW
RC + XTAL	11.49 mW	3.18 mW

^1^ 800 Hz, 3 channels, 24 bit raw data streaming; ^2^ 200 Hz, 1 channel, 20 bit VLDE-encoded data streaming.

**Table 3:** Comparison of the achieved synchronization accuracy with other state-of-the-art approaches.

Ref.	Protocol	Signals	Data Rate	SoC	Accuracy
[25]	2.4 GHz ^1^	generic	1 kb/s	nRF52832	0.8 µs
[21]	BLE	ExG	12 kb/s	CC2640R2F	1 ms ^2^
[21]	BLE	ExG	12 kb/s	nRF52840	1.8 ms ^2^
[23]	BLE	IMU	200 Hz	nRF52832	30 µs
[22,29]	wired ^1^	EMG	1 kHz	0.35 µm ASIC	4.6 ns
[26]	BLE	EMG	57.6 kb/s	nRF52840	6.6 µs ^3,4^
This work	BLE	EMG	57.6 kb/s	nRF52840	47 µs ^3,5^

^1^ Proprietary protocol; ^2^ Referred to the 95th percentile; ^3^ RMS value; ^4^ XTAL oscillator; ^5^ RC oscillator.

## Data Availability

Data are contained within the article.

## Abbreviations

The following abbreviations are used in this manuscript:

ADC	Analog-to-Digital Converter
AFE	Analog Front-End
ASIC	Application-Specific Integrated Circuit
BLE	Bluetooth Low Energy
CPU	Central Processing Unit
LSB	Least Significant Bit
MCU	MicroController Unit
PCB	Printed Circuit Board
PWM	Pulse Width Modulation
RMS	Root Mean Square
THT	Through-Hole Technology
UTC	Coordinated Universal Time
